# The Impact of Locomotor Speed on the Human Metatarsophalangeal Joint Kinematics

**DOI:** 10.3389/fbioe.2021.644582

**Published:** 2021-04-20

**Authors:** Kunyang Wang, Sivangi Raychoudhury, Dan Hu, Lei Ren, Jing Liu, Haohua Xiu, Wei Liang, Bingqian Li, Guowu Wei, Zhihui Qian

**Affiliations:** ^1^Key Laboratory of Bionic Engineering, Ministry of Education, Jilin University, Changchun, China; ^2^School of Mechanical, Aerospace and Civil Engineering, University of Manchester, Manchester, United Kingdom; ^3^School of Science, Engineering and Environment, University of Salford, Salford, United Kingdom

**Keywords:** metatarsophalangeal joint, three-dimensional kinematics, position and orientation, human locomotion, functional axis

## Abstract

This paper aims to further our previous study to investigate the effect of speed on the human metatarsophalangeal (MP) joint kinematics during running on level ground. The 3D motion of the foot segments was captured by a twelve-camera motion analysis system, and the ground reaction forces and moments were recorded by using a six-force plate array. The relative movement between the tarsometatarsi (hindfoot) and phalanges (forefoot) segments were recorded to obtain the 3D orientation and position of the functional axis (FA) of the MP joint. The results show that the FA locates about an average of 19% foot length (FL) anterior to the anatomical axis (AA) across all running speeds, and is also 4.8% FL inferior to the AA during normal and fast run. Similar to walking, the functional axis is more oblique than the anatomical axis with a more anterior–inferior orientation across all the running speeds. This suggests that representing MP joint with the AA may mislead the calculation of joint moment/power and muscle moment arms in both running and walking gait. Compared with previous study, we found that walking and running speeds have statistically significant effects on the position of the FA. The functional axis moves frontward to a more anterior position when the speed increases during walking and running. It transfers upward in the superior direction with increasing speed of walking, but moves more toward the inferior position when the velocity increased further to running. Also, the orientation of FA in sagittal plane became more oblique toward the vertical direction as the speed increased. This may help in moderating the muscular effort, increase the muscle EMA and improve the locomotor performance. These results would contribute to understanding the in vivo biomechanical function of the MP joint and also the foot propulsion during human locomotion.

## Introduction

Human locomotion involves intensive use of the feet to attenuate ground impact, maintain locomotor stability, generate propulsive power across the whole range of the locomotor speed (Ker et al., 1987; Carrier et al., 1994; Ren et al., 2008a; Holowka and Lieberman, 2018). Apparently different activation patterns of foot muscles were observed with increase in moving speed from walking to running (Mann and Hagy, 1979; Minetti et al., 1994; Srinivasan and Ruina, 2006; Lipfert et al., 2012), which lead to distinct ground reaction force (GRF) pattern during walking from during running (Cavanagh and Lafortune, 1980; Nilsson and Thorstensson, 1987, 1989). It was also found that the kinetics and kinematics of the ankle joint and the foot during walking were different from that during running throughout the stance phase (Czerniecki, 1988; Rodgers, 1988; Holowka and Lieberman, 2018). Most previous studies on locomotor biomechanics of the foot focus on the foot segment as whole and/or the ankle joint (Adelaar, 1986; Apkarian et al., 1989; Scott and Winter, 1993; Gefen et al., 2000; Carson et al., 2001; Nester et al., 2007; Altman and Davis, 2012). However, the human metatarsophalangeal (MP) joint which locates at the most distal end of the foot segment may play important roles as well (Bojsen-Møller and Lamoreux, 1979; Mann and Hagy, 1979; Stefanyshyn and Nigg, 1997; Kim et al., 2012).

During human walking, the plantar fascia is gradually stretched up by the dorsiflexion of the metatarsophalangeal joint to wrap around the metatarsal heads (Ker et al., 1987; Fuller, 2000). Accordingly, this action yields some elevation of the longitudinal arch and further stabilise the foot without any muscle actions, benefiting from the windlass mechanism of the plantar aponeurosis (Mann and Hagy, 1979; Fuller, 2000). The moment arm of the ground reaction force could be shortened by the dorsiflexion of the MP joint in the late stance phase acting like a rigid foot lever. Conceivably, the effective mechanical advantage (EMA) of the ankle dorsiflexor muscles will be raised and thereby the muscular effort during push-off be reduced (Bojsen-Møller and Lamoreux, 1979; Biewener, 1989). Additionally, the dorsiflexion of the MP joint may help to constraint the relative skin movements to transmit the shear forces to the skeleton by tightening the connective tissue system in the foot (Bojsen-Møller and Lamoreux, 1979; Vereecke and Aerts, 2008).

As the toes advance to balance the body during rapid changing motions (Mann and Hagy, 1979; Holowka and Lieberman, 2018). The human MP joint maybe features prominently in rapid body movements such as running. One study has found that the MP joint functions as an impressive energy dissipater during sprinting, which absorbs more energy when the speed being increased (Stefanyshyn and Nigg, 1997). Limited dorsiflexion of the MP joint may lead to insufficient energy generation during push-off (Stefanyshyn and Nigg, 1997). Moreover, the literature has presented that the ankle as well as the MP joint may provide a variable gearing mechanism (Carrier et al., 1994) of the ankle dorsiflexor muscles during running. It might improve the locomotor performance during the late stance of running by keeping the muscles working at the high-efficiency region of the force-velocity curve.

The human MP joint has been simplified to a single axis originating from the fifth metatarsal head and perpendicular to the sagittal plane in many studies (Stefanyshyn and Nigg, 1997, 1998; Kim et al., 2012). Though the motion of the MP joint is simplified, this two-dimensional assumption does not express the oblique nature. One study explained the influence of various MP joint axis definitions on calculated variables of joint kinetics in sprinting by using inverse dynamics (Smith et al., 2012). The result showed that the axis definition of the MP joint substantially affects the estimated joint moment, power and energy. The simplified 2D joint axis definition may bring more energy absorption at the MP joint than the oblique joint axes. It suggests that representing the MP joint in a suitable form is crucial to better comprehend the MP joint function during locomotion (Smith et al., 2012). But, almost all previous researches employed simplified definitions of the MP joint purely on the basis of anatomical landmarks on the foot, e.g., a straight line linking the 1st and 5th metatarsal heads (Bojsen-Møller and Lamoreux, 1979; Smith et al., 2012; Zelik et al., 2015). Until now, the three-dimensional orientation and position of the actual functional rotation axis of the MP joint during human locomotion and how does it change along different locomotor speeds have not been studied thoroughly. In our most recent study, the position and orientation of the functional axis (FA) have been identified in walking with various speeds (Raychoudhury et al., 2014). It was found that FA shows a statically different position and orientation from the anatomical axis (AA) which is defined as a straight line connecting the first and fifth metatarsal heads, and the speed of walking has statically significant effect on the functional axis positions.

This paper aims to further our previous research to investigate the impact of locomotor speed on the human MP joint kinematics. The position and orientation of the FA axis of the MP joint in the stance phase of running at different speeds were determined by the 3D motion capture system. Statistical analysis has been performed to assess the difference between the functional axis of the human MP joint and the anatomical axis typically used in literature. Moreover, by integrating with the walking dataset obtained in our previous study, the impact of locomotor speed on the orientation and position of the human MP joint was statistically analyzed. This would further reveal the underlying mechanism of the MP joint function and also the foot propulsion during locomotion at various speeds.

## Materials and Methods

### Gait Measurement

The experiment procedure was the same with our previous study (Raychoudhury et al., 2014). Six healthy adults with normal foot (*N* = 6, all males; age 26.67 ± 2.69 years; mass 67.17 ± 10.29 kg; height 1.75 ± 0.04 m; mean ± SD), with no previous medical history of lower limb and foot injury, participated in this study. These six participants were the same subjects involved in the walking trials (Raychoudhury et al., 2014). The main reason to take the same subjects was to statistically compare the results of running with walking. These subjects were previously provided written informed consent before participation and separately provided written consent in accordance with the policies of the ethical committee of the university. They were asked to run barefoot on the walkway with self-selected slow (2.51 ± 0.26 m/s), normal (3.12 ± 0.22 m/s) and fast (4.14 ± 0.65 m/s) speed. The same marker cluster system was mounted on the right feet to record the relative motion of the foot. 3D segmental motions were measured by the twelve infrared 3D motion capture system (Qualisys, Sweden) at 150 Hz. The GRF and the moment were measured at 1,000 Hz by a six-force-plate array (Kistler, Switzerland) to only determine the gait cycle. The calibration process was the same as in the walking trials (Raychoudhury et al., 2014). Cappozzo et al. (1995) suggested that if the anatomical landmarks on the bone could been directly measured (such as intracortical pin markers that are drilled in the bones or biplane X-ray imaging system), the relevant calibration parameters are readily available. Otherwise, if the markers are attached on the skin (in this paper), the calibration procedure (CAST) should be conducted. Under each condition of the different running speed, the representative running data were ensured by repeating 15 times of the gait measurement.

### Definition of the Joint Parameters

**FIGURE 1:**
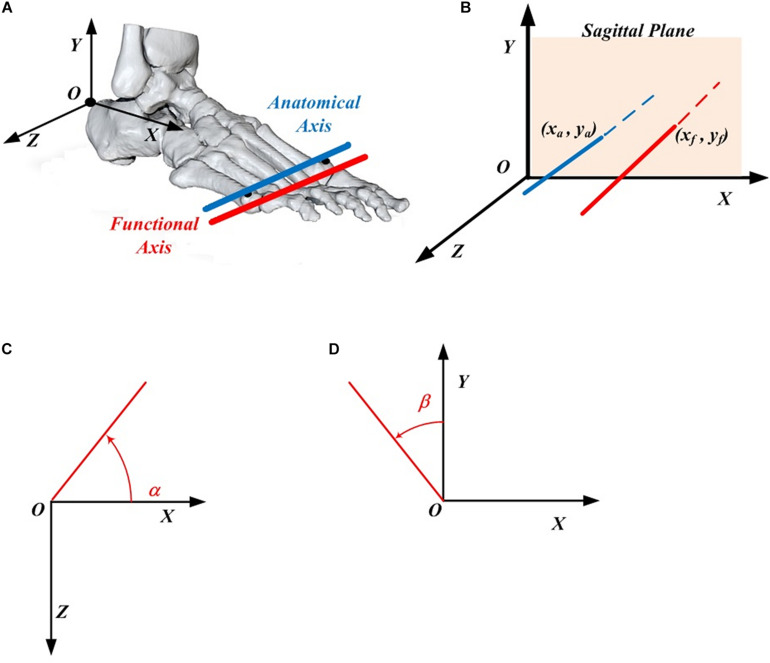
Definition of the MP joint parameters. **(A)** The anatomical axis (blue) and functional axis (red) of the MP joint. **(B)** The position of the functional axis (*x*_*f*_, *y*_*f*_) and the anatomical axis (*x*_*a*_, *y*_*a*_) in the foot coordinate system. **(C)** Orientation angle α made by the functional (or anatomical) axis with respect to the *X* axis in the *XOZ* plane. **(D)** Orientation angle β made by the functional (or anatomical) axis with respect to the *Y* axis in the *XOY* plane.

The whole lower limb was divided into five rigid segments: phalanges, tarsometatarsi, shank, thigh and pelvis according to the literature (Kim et al., 2012; Zelik et al., 2015; Holowka and Lieberman, 2018). The anatomical coordinate system for each mentioned segment was defined based on the previous studies (Ren et al., 2008a, 2010). There were few assumptions made for this study. First, the five phalanges formed a single rigid segment, although there are some relative movements between the phalanges during motion. Second, the MP joint was considered to be a single hinge joint. As shown in Figure 1A, the AA was described as the line linking the 1st and 5th metatarsal heads and the functional axis was defined as the rotational axis between the hindfoot and forefoot, with more details described in Raychoudhury et al. (2014). The closed-form algorithm (Gamage and Lasenby, 2002) was adopted to evaluate the 3D position and orientation of the functional axis.

The 3D positions of FA (*x_*f*_, y_*f*_*) and AA (*x_*a*_, y_*a*_*) are described in the *XOY* plane of the foot local coordinate system (Figure 1B), in which the origin locates at the upper ridge of the calcaneus bone (Ren et al., 2010). The 3D orientations of FA and AA are described as the projected angle in the *XOZ* (α) and *XOY* (β) plane of the foot local anatomical coordinate system. Based on the right-hand rule, the positive angle α is quantified arising from the *X* axis in the counter-clockwise direction, whereas the negative angle β was quantified arising from the *Y* axis in the clockwise direction (Figures 1C,D).

### Data Analysis

GMAS (Generalised Motion Analysis Software), a MATLAB based package for 3D kinematic and kinetic analysis (Ren et al., 2005, 2008b), was used to process the raw data including the locomotion speed. Trials with over 10 consecutive missing frames were dismissed. Gap-filling process was also completed in GMAS. After that, the data was filtered by applying a low pass zero lag fourth-order Butterworth filter with a cut-off frequency of 6.0 Hz. The 6 Hz for filter was used in post-processing to reduce the noise and smooth the raw data. This acquisition was defined according to the max numbers of the gait cycles that participants can performed in one second, in another word, to max speed divided by step length. The fast running speed is less than 4.8 m/s in this study and the step length is around 0.8–1.0 m, so the cut-off frequency was set to 6 Hz. For each running speed, means and standard deviations of position and orientation were calculated across subjects.

Since the foot size differs with subjects, the position parameters *x* and *y* for both the FA and AA were normalised by the foot length of each subject (determined as the displacement from the origin to the mid-point of the 1st and 5th metatarsal heads). The effect of speed on the FA was investigated, in which the data from the previous paper were also taken into consideration, to compare the statistical differences between each pair of speeds and to study the trend change from slow walk to fast run. Since the leg length was different for each subject, the velocity was also normalised as below to quantify the change in the position parameter of the FA in the anterior–posterior direction,

(1) $$V=\frac{\nu }{\sqrt{\text{g}\,L}}$$

where *v* is the velocity of one gait cycle that equals to *dx*/*T* (*dx* is the displacement of the origin from one heel-strike to the next heel-strike in the *X* axis, and *T* is defined as the number of frames between the two heel-strikes divided by the sample frequency of 150 Hz), g is the gravitational constant with the value of 9.816 m/s^2^, and *L* is the leg length of each subject from the hip joint centre to the ground.

#### Statistical Analysis

The statistical analysis was performed to evaluate the difference between the 3D position and orientation of the functional axis and anatomical axis of the MP joint during running, as well as the effect of speed on the FA, from slow walk to fast run using SPSS 20.0 software (IBM, Armonk, NY, United States). For each condition, means and standard errors of position and orientation were calculated across subjects, with standard error indicating inter-subject variability. Similar to the previous study (Raychoudhury et al., 2014), all the position and orientation parameters (*x, y* and α, β) were analyzed separately using the analysis of variance (ANOVA) (random effects: subjects and trials; fixed effects: axis types and the six speeds). We used Fisher’s least significant difference (LSD) multiple comparison to investigate the difference between the two axes and between each pair of speeds. These comparisons were obtained from the least-squared means method, considering the probability *p* < 0.05 as statistically significant.

## Results

**FIGURE 2:**
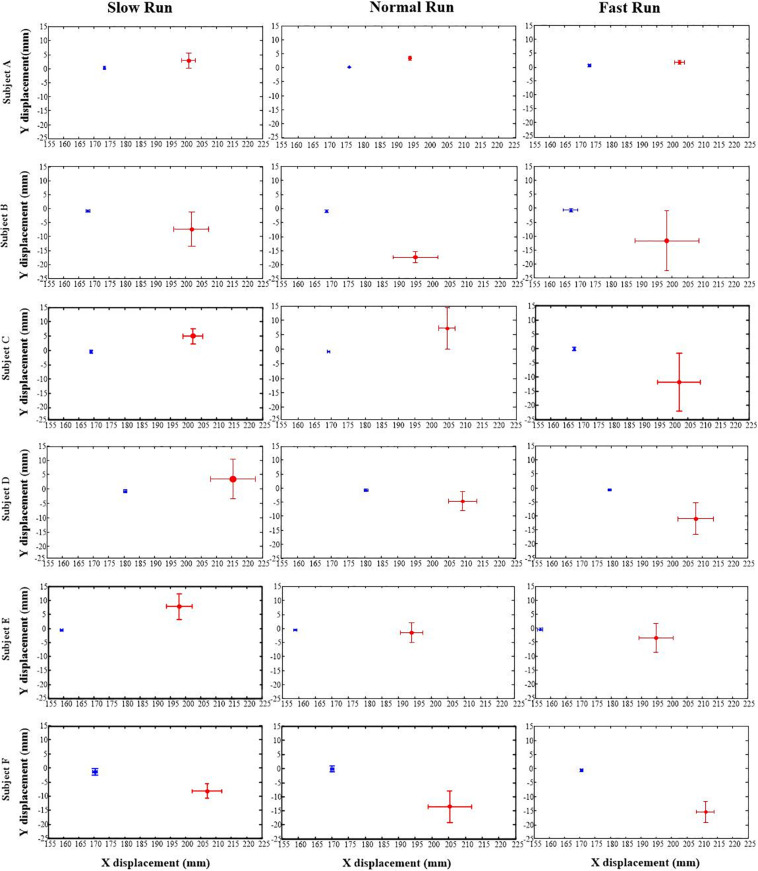
The positions of the anatomical axis (blue) and the functional axis (red) of the MP joint for all the six subjects (A–F) across all the three speeds of running (slow, normal, fast). Solid dots depict mean; bars depict the 1 SD zones.

Figure 2 depicts the position data of the AA (in blue) and FA (in red) of the MP joint for all the six subjects under all the three different running speeds (slow, normal and fast). In general, the AA was found to be consistently posterior to the FA for all the subjects and for all the running speeds, with an average position difference of 32.58 mm. The result shows that the position of the FA in *Y* axis is moving downward with increasing speed of running. Meanwhile, in the anterior–posterior direction (along *X* axis) the FA during normal and fast run does not show any change, but with slow running speed, it presents a very little backward movement yet staying anterior to the AA. In comparison with FA, AA shows no distinctive change in the position along all the directions with increasing speed for all the subjects.

**FIGURE 3:**
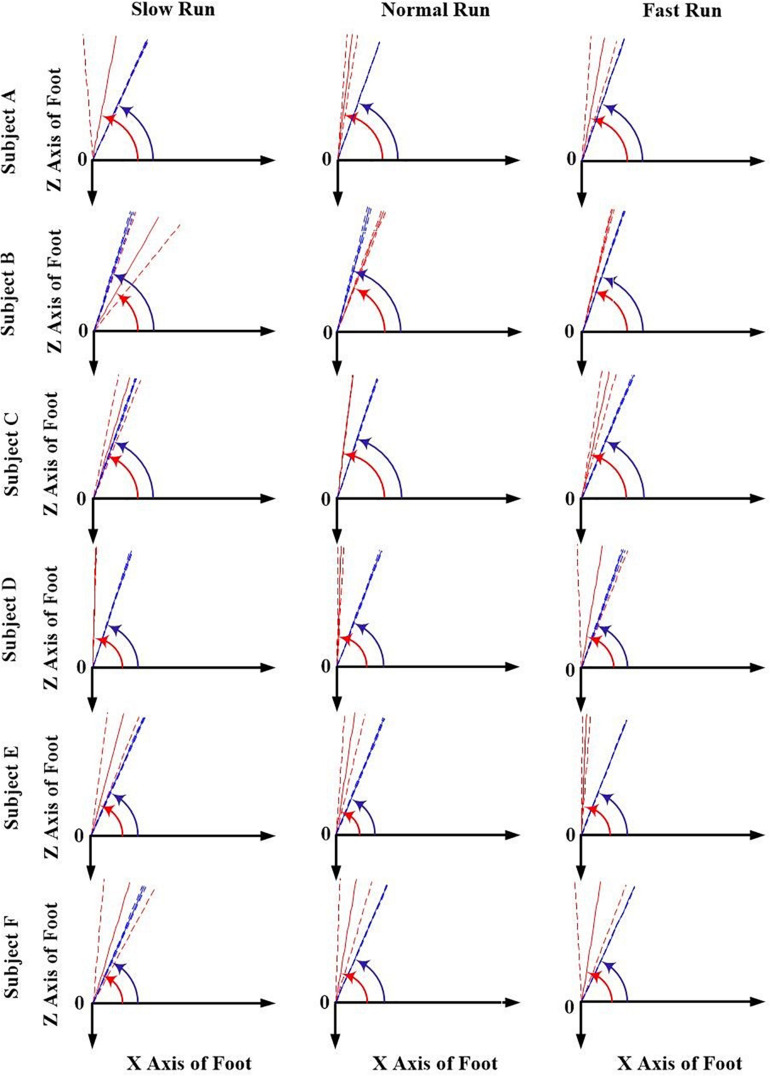
The orientation angle α made by the anatomical axis (blue) and functional axis (red) for all the six subjects (A–F) across all the three speeds of running (slow, normal, fast). Solid lines depict mean; dashed lines depict the 1 SD zones.

**FIGURE 4:**
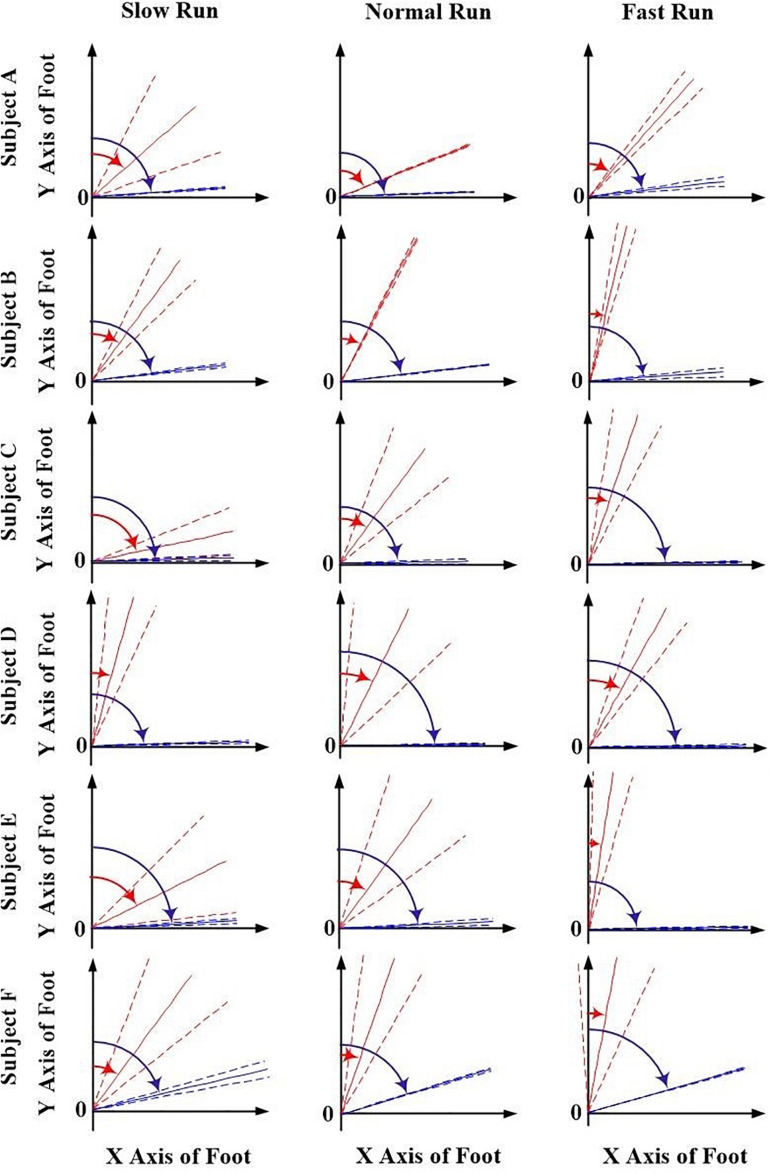
The orientation angle β made by the anatomical axis (blue) and functional axis (red) for all the six subjects (A–F) and across all the three speeds of running (slow, normal, fast). Solid lines depict mean; dashed lines depict the 1 SD zones.

The orientation parameters, angle α and β of both the FA (red) and AA (blue) along all the running speeds (slow, normal and fast), were presented in Figures 3, 4. Generally, in the transverse plane, the orientation of the FA of the MP joint was more inclined (greater angle α) to the *Z* axis than that of AA for all the subjects, except subject B during slow and normal run (Figure 3). But, no obvious change in the obliquity of both the axes (FA and AA) with the increasing running speed was observed. From the Figure 4, it suggests that for all the subjects the FA showed a very different orientation than that of AA in the sagittal plane across all running speeds. The functional axis possessed higher angle β than the anatomical axis, causing a more inclined orientation to the *Y* axis, and this inclination increased from slow to fast run. On the other hand, the orientation angle β in the sagittal plane for AA remained few differences when the speed changed.

The statistical analysis results of the 3D orientation and position differences between the FA and AA were described in Table 1. We found that except the position parameter *y* at slow run, statistically significant differences existed in all the parameters (*x, y*, α, β) across all the three running speeds. During normal and fast run, the FA located more anteriorly and inferiorly than AA, which is significant in both directions (vertical and horizontal). Furthermore, the difference of the orientation angle was less significant in the transverse plane than that in the sagittal plane.

**TABLE 1:** Statistical analysis of the 3D orientation and position differences between the FA and AA of the MP joint during running.

		***x* (normalised by foot length)**	***y* (normalised by foot length)**	**α (degrees)**	**β (degrees)**
Slow run	FA	1.20 ± 0.04^a^	0.010 ± 0.040^a^	78.64 ± 8.90^a^	−48.91 ± 26.65^a^
	AA	0.99 ± 0.01^b^	−0.003 ± 0.004^b^	73.21 ± 2.55^b^	−85.56 ± 4.89^b^
Normal run	FA	1.18 ± 0.04^a^	−0.020 ± 0.050^a^	82.93 ± 5.48^a^	−38.38 ± 22.69^a^
	AA	0.99 ± 0.01^b^	−0.002 ± 0.003^b^	72.40 ± 2.34^b^	−85.87 ± 5.46^b^
Fast run	FA	1.18 ± 0.04^a^	−0.050 ± 0.050^a^	82.00 ± 5.94^a^	−24.84 ± 17.50^a^
	AA	0.99 ± 0.01^b^	−0.001 ± 0.004^b^	72.47 ± 2.36^b^	−86.18 ± 5.38^b^

*The value of mean ± SEM for all the trials across all the subjects were depicted. Different letters mean that the variable in a column differs significantly with each other (*p* < 0.05).*

As shown in Table 2, another statistical analysis was conducted to study the impact of speed on the 3D position and orientation of FA. In this study, the running data were compared with the walking data (Raychoudhury et al., 2014) and some significant differences were found from walking to running. Here, the pairwise comparison was taken between each individual speed. The results indicate that the position of the FA in the *X* axis showed statistically significant difference when the speed was increased from walking to running. Moreover, with the speed increasing from slow walk to slow run, a forward shift of the FA toward anterior direction was also observed. But, when the speed was further increased to normal and fast run, the position of FA in the anterior–posterior direction was found to move little backward. No significant change was observed when the speed increases to running except for the normal run, which showed statistically significant difference between slow and normal run.

**TABLE 2:** Statistical analysis of the effect of the increasing speed from walking to running on the position and orientation of the functional axis (FA) of the MP joint.

	***x* (normalised by foot length)**	***y* (normalised by foot length)**	**α (degrees)**	**β (degrees)**
Slow walk	1.15 ± 0.04^ab^	0.004 ± 0.020^abd^	77.93 ± 8.08^a^	−52.40 ± 25.26^*abcd*^
Normal walk	1.16 ± 0.04^abce^	0.020 ± 0.020^abcd^	81.26 ± 6.43^ab^	−51.07 ± 15.03^abcd^
Fast walk	1.17 ± 0.04^bce^	0.030 ± 0.030^bc^	80.05 ± 6.35^ab^	−47.20 ± 23.47^*abcde*^
Slow run	1.20 ± 0.04^df^	0.007 ± 0.040^abd^	78.64 ± 8.90^ab^	−48.91 ± 26.65^abcde^
Normal run	1.18 ± 0.04^bcef^	−0.020 ± 0.050^e^	82.93 ± 5.48^b^	−38.38 ± 22.69^*cde*^
Fast run	1.18 ± 0.04^def^	−0.050 ± 0.050^f^	82.00 ± 5.94^ab^	−24.84 ± 17.50^f^

*The value of mean ± SEM for all the trials across all the subjects were depicted. Different letters mean that the variable in a column differs significantly with each other (*p <* 0.05).*

More significant changes were observed in the *Y* direction when the speed increased gradually from slow walk to fast run. In our previous study (Raychoudhury et al., 2014), the FA started moving toward more superior position when the speed increased from slow walk to fast walk. Interestingly, when the speed was increased further to slow run followed by normal run and fast run, the FA was observed to move downward, toward more inferior position. Significant differences were found in the normal and fast runs, where the FA moved even inferior to the origin. We found no statistically significant effects on the angle α of the FA in the transverse plane when the speed was increased from walking to running, except for the slow walk and normal run. However, more significant effects were found in the FA orientation angle β in the sagittal plane. The orientation of the FA changed greatly during the fast running speed, which showed a statistically significant difference from the other five speeds. From slow walk to slow run, no significant effects were found in this orientation. And normal run showed statistically significant difference between the first two speeds of walking and fast run. Generally, when the speed increased gradually, the FA became more obliquely oriented in the sagittal plane.

**FIGURE 5:**
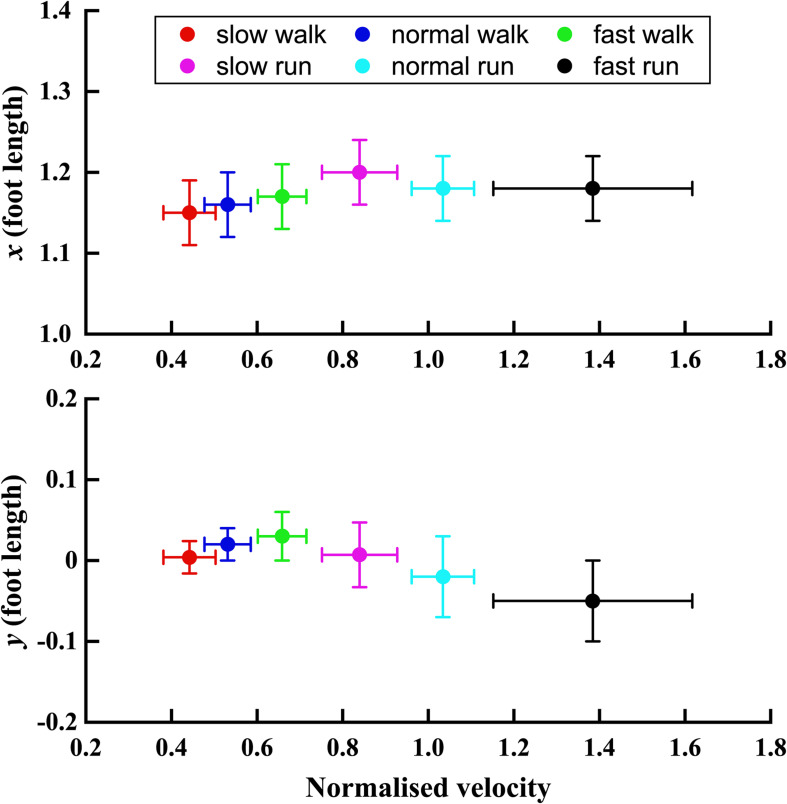
The trend of *x* and *y* positions (normalised by the foot length) of the FA with the normalised velocity *V* increasing from slow walk to fast run. Circle plots depict mean; bars depict the 1 SD zones.

In Figure 5, the trend of the position parameter *x* and *y* normalised by the foot length was shown with normalised velocity *V*. It can be seen that the *x* position of the FA of the MP joint shifts forward till slow run, and then for normal run and fast run the axis shifted backward with no difference between the last two running speeds. The normalised position parameter *y* moved upward in the superior direction during slow, normal and fast walk. But, when the velocity increased further, the FA’s *y* position moved more toward the inferior direction. Also, it shifted below the origin during normal and fast run.

**FIGURE 6:**
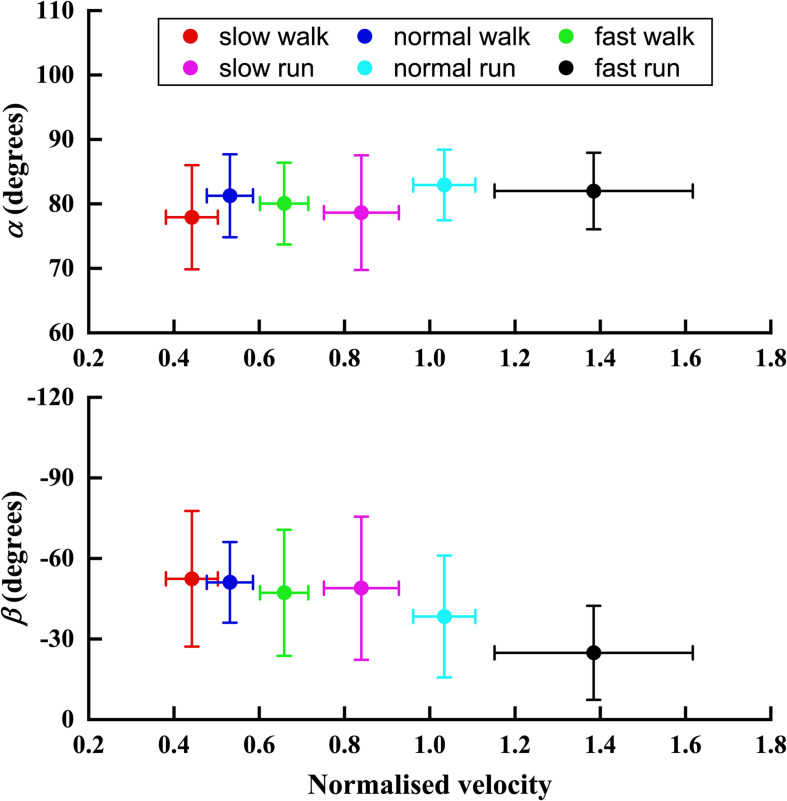
The trend of the orientation angle α and β of FA with the normalised velocity *V* increasing from slow walk to fast run. Circle plots depict mean; bars depict the 1 SD zones.

Figure 6 depicted the trend of the orientation angle α and β of the FA of MP joint in the sagittal plane, with the increasing normalised velocity *V* from slow walk to fast run. In the transverse plane, no obvious trend was found in the angle α. However, unlike angle α, the orientation angle β showed an obvious trend with increasing speed. Namely, as the normalised speed increased, the orientation of FA in sagittal plane became more oblique toward the *Y* axis.

## Discussion

The study aims to extend the research of 3D position and orientation of the human MP joint from walking to running gait. Similar as walking, the 3D orientation and position of the FA during running is close to the AA, and statistically significant differences have been observed between the functional axis and anatomical axis. The FA remained anterior to the AA for all the running speed conditions with the average distance of about 19% FL. Compared with walking, the anterior–posterior position of the FA has shifted forward by 3% during running. In the vertical direction, the FA moved about 4.8% FL toward the inferior position than AA during normal and fast run. In contrast with the walking data, the FA during running moved downward, even below the origin of the local foot coordinate system. Also, the FA shows higher obliquity than AA across all the running speeds with anteriorly inferior orientation. This indicates that the representing the MP joint with the AA may lead to incorrect joint moment/power estimation and inaccurate moment arm calculation, especially for running.

Statistically significant effects were found on the position and orientation of the FA when the speed gradually increased from walking to running. Similar to walking, when the speed increased to running, the FA shifted itself toward more anterior position. On the other hand, with the speed increase in running, the FA shifted toward inferior position (superior in walking). In other words, this MP joint axis displayed a forward movement toward the ground reaction force vector during the late stance phase, when the speed increased from walking to running. It was also observed that the orientation of the FA became more oblique as the running speed increased. As a result, the moment arm of the GRF decreased with the moment arm of the MP extensor muscles being increased simultaneously, and this inversely relation would be magnified with the increasing speed. Accordingly, the increase of the effective mechanical advantage (EMA) of the corresponding muscles (Biewener, 1989, 1990, 2003; Biewener et al., 2004) may reach to the maximum during fast run. In the same way, the increased lever distance to the ankle joint benefited from the forward shift of FA will be enlarged with the increasing speed from walking to running. All of these may provide the MP extensor muscles contracting in the optimal power region with a proper velocity (Biewener, 2003). Furthermore, the results suggest that the MP joint also functions like a variable gear especially during running (Carrier et al., 1994), which will improve the locomotor performance by keeping the MP extensor muscles working at the high-efficiency region of the force-velocity curve.

There were some limitations of this research. First of all, the movement between the hindfoot and the forefoot throughout the stance phase was represented by the single hinge type. The option of a single axis for MP joint forgot that the first toe is of crucial importance in the propulsive phase in human locomotion and most of the times have a different axis orientation in relation with the other four toes. More attention should be payed to this in future studies. Second, the phalanges and the hindfoot segments were considered as individual single rigid segments. However, there may be some relative movements present between the phalanges or the hindfoot bones. Third, only 6 adults participated in this study, and we will involve more participants and do power analysis for further research. Besides, there may be some skin artefacts present due to the skin markers involved in the gait measurement. In future, the biplane X-ray imaging system would be used to quantify the joint motion.

## Conclusion

yIn this study, we statistically evaluated the difference between the functional axis of the human MP joint and the anatomical axis typically used in literature during running with different speeds. The 3D oblique orientation of the functional axis along with its relative position in the foot coordinate system were presented. The position of the FA locates anterior and superior to the AA in a more oblique orientation during running, same as walking. Statistically analysis has been performed to study the impact of locomotor speed ranging from slow walk to fast run on MP joint position and orientation, by integrating with the walking dataset obtained in our previous study. With the increasing speed of locomotion, the joint axis shifts forward in anterior–posterior direction, but in the superior–inferior direction it first moves upward and then shifts downward. The increase of the moment arm of the MP extensor muscles and decrease of the moment arm of the GRF may help moderate the muscular effort of MP extensor muscles, increase the muscle EMA, and improve the locomotor performance. These findings would further understand the underlying mechanism of the in vivo biomechanical function of the metatarsophalangeal joint during locomotion. Moreover, they might enhance the bionic designs of the robotic legs, therapeutic footwear, and even prosthetic lower limbs.

## Data Availability Statement

The raw data supporting the conclusions of this article will be made available by the authors, without undue reservation.

## Ethics Statement

The studies involving human participants were reviewed and approved by University’s Ethical Advisory Committee. The patients/participants provided their written informed consent to participate in this study.

## Author Contributions

LR, ZQ, and GW conceived this research. KW, SR, and LR performed the experiments and drafted the manuscript. DH, JL, HX, WL, and BL were responsible for data processing, discussions and revisions. All authors contributed to the article and approved the submitted version.

## Conflict of Interest

The authors declare that the research was conducted in the absence of any commercial or financial relationships that could be construed as a potential conflict of interest.
